# Risk factors and prognosis of poor graft function after allogeneic hematopoietic stem cell transplantation in pediatric: a retrospective study

**DOI:** 10.3389/fcell.2025.1651658

**Published:** 2025-10-08

**Authors:** Guanxiu Pang, Xiaobo Wang, Wenguang Jia, Mengchen Li, Tianyuan Zhou, Jianming Luo, Yunyan He

**Affiliations:** ^1^ Department of Pediatrics, The First Affiliated Hospital of Guangxi Medical University, Nanning, China; ^2^ The First Affiliated Hospital of Guangxi Medical University / Difficult and Critical Illness Center, Pediatric Clinical Medical Research Center of Guangxi, Nanning, China; ^3^ Department of Hematology, The Seventh Affiliated Hospital of Sun Yat-sen University, Shenzhen, China

**Keywords:** children, allogeneic hematopoietic stem cell transplantation, poor graft function, risk factors, prognosis

## Abstract

**Introduction:**

Poor graft function (PGF) represents a serious and potentially life-threatening complication following allogeneic hematopoietic stem cell transplantation (allo-HSCT); however, its etiological risk factors and prognostic implications remain inadequately defined within pediatric populations.

**Methods:**

A retrospective cohort study was conducted on 175 pediatric patients undergoing allo-HSCT between 30 June 2018, and 31 December 2022. Patients were stratified into PGF (n = 30) and good graft function (GGF, n = 145) groups. Multivariate logistic regression identified risk factors for PGF, while Cox proportional hazards models evaluated mortality-associated variables. Survival outcomes were analyzed using Kaplan-Meier curves.

**Results:**

Key findings encompass: (1) PGF Risk Factors: Multivariable analysis identified four independent predictors of PGF: age ≥10 years at transplantation (OR = 29.27, 95%CI: 5.70–150.21, P < 0.001), HLA mismatch (OR = 4.11, 95%CI: 1.45–11.65, P = 0.008), cytomegalovirus (CMV) infection (OR = 7.64, 95%CI: 2.31–25.21, P = 0.001), and BK virus (BKV) infection (OR = 12.22, 95%CI: 2.49–59.89, P = 0.002); The model’s predictive performance by ROC analysis yielded an AUC of 0.886 (95%CI: 0.83–0.94; P < 0.001). (2) Survival Analysis: the 4-year overall survival (OS) was profoundly inferior in the PGF cohort compared to the GGF cohort (49.4% ± 10.3% vs. 90.2% ± 2.5%, P < 0.001). (3) Predictors of Mortality: Cox regression identified PGF (HR = 2.39, 95%CI: 1.02–5.59, P = 0.044), acute graft-versus-host disease (grade I/II, HR = 3.43, 95%CI: 1.29–9.15, P = 0.014; grade III/IV, HR = 8.92, 95%CI: 3.19–24.96, P < 0.001), hemorrhagic cystitis (HR = 3.18, 95%CI: 1.37–7.39, P = 0.007), and severe pneumonia (HR = 4.42, 95%CI: 1.92–10.19, P < 0.001) as independent predictors of early mortality.

**Conclusion:**

Age ≥10 years at transplantation, HLA mismatch, CMV infection, or BK viremia identifies a high-risk cohort of pediatric allo-HSCT recipients who require intensified monitoring for PGF, underscoring an urgent need for effective preventive and therapeutic interventions.

## Introduction

Allogeneic hematopoietic stem cell transplantation (allo-HSCT) remains a definitive treatment for hematologic malignancies, marrow failure syndromes, and inherited disorders. Despite achieving long-term remission in over 40,000 annual recipients globally (Passweg et al., 2021), poor graft function (PGF)—characterized by full donor chimerism (≥95%) with sustained multilineage cytopenia (neutrophils <0.5 × 10^9^/L, platelets <20 × 10^9^/L, hemoglobin <70 g/L) —confers substantial mortality risks through hemorrhagic complications and opportunistic infections (Prabahran et al., 2022; Kong, 2019). Clinically, PGF manifests as either primary (failed engraftment by day +28) or secondary (cytopenias post-initial engraftment) subtypes (Kong, 2019; Sun et al., 2015; Zhao et al., 2019). Epidemiological analyses reveal cumulative incidence rates of 5%–27% (Dominietto et al., 2001; Man et al., 2022), with primary and secondary PGF affecting 1.5%–12.1% and 12.7%–16.3% of recipients (Zhao et al., 2019; Xiao et al., 2014), respectively. Prognostically, primary PGF was associated with markedly reduced overall survival (OS: 25%–34.6% at 1 year; 6% at 2 years), whereas secondary PGF shows partial hematopoietic recovery (53.6%) but limited survival benefit compared to those with GGF (5,9,10), highlighting the urgent need for improved management strategies.

Contemporary research identifies multiple peritransplant risk modifiers for PGF development, including but not limited to splenic enlargement (Zhao et al., 2019; Chen et al., 2022), elevated pretransplant serum ferritin levels (Zhao et al., 2019; Taoka et al., 2012), HLA mismatch (Sun et al., 2015; Taoka et al., 2012), ABO incompatibility (Xiao et al., 2014; Chen et al., 2022), low CD34^+^ cell doses (Zhao et al., 2019; Sun et al., 2019), and cytomegalovirus (CMV) reactivation episodes (Lv et al., 2021; Lin et al., 2022). Nevertheless, existing evidence remains constrained by heterogeneous findings across studies and a paucity of pediatric-specific data. This investigation proposes to conduct a comprehensive retrospective cohort analysis of pediatric allo-HSCT recipients, employing multivariate regression models to systematically evaluate modifiable risk parameters of PGF. The ultimate objectives encompass refinement of transplantation protocols, implementation of risk-adapted preventive strategies, and consequent improvement in long-term survival metrics for this vulnerable patient population.

## Materials and methods

### Patients

A retrospective analysis was conducted on the clinical data of 175 pediatric patients who underwent allo-HSCT at the Pediatric Transplant Center of the First Affiliated Hospital of Guangxi Medical University between 30 June 2018, and 31 December 2022. Inclusion criteria: Recipients undergoing their first allo-HSCT and their respective donors, age under 18 years, and with guardians having signed consent forms acknowledging transplant-related risks. Exclusion criteria: Incomplete clinical data. Finally, a total of 175 patients met these criteria and were enrolled in the study. The study was reviewed and approved by the ethics committees of The First Affiliated Hospital of Guangxi Medical University.

### Data collection

Demographic characteristics of recipients (sex, age), primary disease type, splenomegaly status, splenectomy history, pre-transplant serum ferritin levels, donor-recipient matching parameters (sex, blood type, HLA matching), graft source, conditioning regimens, transplantation approach, CD34^+^ cell dose (x 10^6^/kg), total nucleated cell dose (x 10^6^/kg), as well as neutrophil and platelet engraftment times were systematically documented. Post-transplant complications data encompassing hemorrhagic cystitis, secondary hypertension, secondary hyperglycemia, acute graft-versus-host disease (aGVHD), chronic graft-versus-host disease (cGVHD), CMV infection, Epstein-Barr virus (EBV) infection, BK virus (BKV) infection, and severe pneumonia were stratified based on temporal occurrence relative to PGF development and OS endpoints. The primary endpoint was OS duration, calculated from transplantation date until death or last follow-up. Secondary endpoints included PGF incidence rates and time-to-PGF, defined as the interval between hematopoietic stem cell infusion and PGF diagnosis. All patients were followed through 31 December 2023.

### Transplantation conditioning regimen

All patients underwent conditioning regimens to eradicate abnormal clones and disrupt disease mechanisms. The regimens varied based on patient tolerance and disease status, with 10 specific combinations listed (Supplementary Table S1).

### Post-transplant complications management

The basic GVHD prevention regimen consists of cyclosporine A and mycophenolate mofetil (Penack et al., 2020; Brown et al., 2020). For haploidentical donor transplants, additional post-transplant cyclophosphamide is used on days +3 and +4 for prevention (M et al., 2016; Anurathapan et al., 2016). The GVHD grading system follows the modified Glucksberg criteria and the international consensus grading system (Schoemans et al., 2018; Stem Cell Application Group, Hematology Branch of Chinese Medical Association, 2020). Other preventive measures include appropriate antibiotic use during conditioning to prevent infections, the administration of low-molecular-weight heparin, prostaglandin E1, and ursodeoxycholic acid to prevent transplant-associated hepatic veno-occlusive disease, proper hydration and alkalinization to prevent hemorrhagic cystitis, and oral phenytoin to prevent reversible posterior leukoencephalopathy syndrome.

### Treatment of PGF

All patients diagnosed with PGF received standardized supportive care comprising blood product transfusions, anti-infective prophylaxis, and intravenous immunoglobulin administration for immunomodulatory support. First-line cytokine therapy comprised subcutaneous granulocyte colony-stimulating factor combined with thrombopoietin receptor agonists (TPO-RAs, e.g.,,eltrombopag). For refractory cases, cellular therapies were initiated: donor lymphocyte infusion at escalating doses (1 × 10^6^ to 1 × 10^7^ CD3^+^ cells/kg), CD34^+^ stem cell boosts (>2 × 10^6^ CD34^+^ cells/kg), and third-party mesenchymal stromal cell (MSC) infusions (1–2 × 10^8^ viable cells/kg/dose administered every 14 ± 2 days for 2-3 cycles) to facilitate hematopoietic niche reconstitution.

### Statistical methods

Statistical analyses were performed using SPSS 26.0 software (IBM, United States). Continuous variables with normal distribution are expressed as mean ± standard deviation and were compared using independent samples t-tests. Non-normally distributed continuous data are summarized as median (interquartile range) and compared using the Mann–Whitney U test. Categorical variables are presented as number (percentage) and analyzed with the Pearson χ^2^ test or Fisher’s exact test, as appropriate. Multivariable logistic regression was used to identify independent risk factors for PGF. The discriminative ability of the regression model was assessed using receiver operating characteristic (ROC) curve analysis. Survival distributions were estimated by the Kaplan-Meier method and compared with the log-rank test. A two-sided p-value <0.05 was considered statistically significant.

## Results

### Incidence and characteristics

Among 175 patients, neutrophil engraftment was achieved in 172 (98.3%), and platelet engraftment was achieved in 162 (92.3%). The mean time to neutrophil engraftment was 14.0 ± 0.40 days, while the median time to platelet engraftment was 16.0 days (range, 6–89 days). The median infused CD34^+^ cell dose was 6.65 × 10^6^/kg (range, 0.84–40.77 × 10^6^/kg). 3 patients failed to achieve engraftment of both neutrophils and platelets by day 28 and were diagnosed with primary PGF.

By 31 December 2023, PGF occurred in 30 patients (17.1%) among the 175 analyzed cohort, while GGF was observed in 145 patients (82.9%). Of the PGF cases, 3 (10.0%) were classified as primary PGF and 27 (90.0%) as secondary PGF. Comparative analysis revealed that PGF patients were significantly older at transplantation (mean age 9.8 ± 0.7 years vs. 6.5 ± 0.3 years; P < 0.001) and exhibited higher rates of splenectomy (43.3% vs. 16.6%; P = 0.001). Additionally, the PGF cohort demonstrated greater prevalence of malignant comorbidities (26.7% vs. 10.3%; P = 0.016), increased HLA mismatch frequency (50.0% vs. 27.6%; P = 0.016), and higher incidence of post-transplant complications, including BKV infection (26.7% vs. 4.8%; P < 0.001), hemorrhagic cystitis (56.7% vs. 31.7%; P = 0.010), severe cGVHD (10.0% vs. 2.0%; P = 0.025) and severe pneumonia (26.7% vs. 11.0%; P = 0.048) (Table 1).

**TABLE 1:** Clinical characteristics of the PGF and GGF patients.

Characteristics	PGF (n = 30)	GGF (n = 145)	P Value
Disease n (%)			0.102
Thalassemia	14 (46.7)	104 (71.7)	
AA	4 (13.3)	14 (9.7)	
AL	5 (16.7)	14 (9.7)	
HLH	2 (6.6)	5 (3.4)	
others	5 (16.7)	8 (5.5)	
Gender n (%)			0.581
Male	17 (57.1)	90 (62.1)	
Female	13 (42.9)	55 (37.9)	
Age (years, mean ± SD)	9.8 ± 0.7	6.5 ± 0.3	<0.001
SF level (ug/L, median, range)	3027.1 (66.7–15151.0)	2751.9 (35.9–14463.1)	0.765
Splenomegaly			0.001
Yes	13 (43.3)	24 (16.6)	
No	17 (56.7)	121 (83.4)	
Types of primary onset n (%)			0.035
Malignant	8 (26.7)	15 (10.3)	
Non-malignant	22 (73.3)	130 (89.7)	
Blood mismatch n (%)			0.279
Identical	15 (50.0)	88 (60.7)	
Mismatch	15 (50.0)	57 (39.3)	
HLA disparity n (%)			0.016
Matched	15 (50.0)	105 (72.4)	
Mismatched	15 (50.0)	40 (27.6)	
Source of stem cell n (%)			0.084
BM	4 (13.3)	15 (10.4)	
BM + UCB	3 (10.0)	35 (24.1)	
BM + PB	21 (70.0)	94 (64.8)	
PB	2 (6.7)	1 (0.7)	
Neutrophil recovery (days, mean ± SD)	17.1 ± 0.8	17.0 ± 0.5	0.936
Platelet recovery (days, median, range)	19.0 (8.0–89.0)	17.0 (8.0–32.0)	0.017
CD34^+^ cell dose (10^6^/kg, median, range)	5.5 (2.0–34.3)	7.10 (0.8–40.8)	0.485
CMV infection n (%)			0.108
Yes	21 (70.0)	120 (82.8)	
No	9 (30.0)	25 (17.2)	
EBV infection n (%)			0.236
Yes	14 (46.7)	51 (35.2)	
No	16 (53.3)	94 (64.8)	
BKV infection n (%)			<0.001
Yes	8 (26.7)	7 (4.8)	
No	22 (73.3)	138 (95.2)	
Hemorrhagic cystitis n (%)			0.010
Yes	17 (56.7)	46 (31.7)	
No	13 (43.3)	99 (68.3)	
aGVHD n (%)			0.450
No	22 (73.3)	120 (82.8)	
Grade I-II	5 (16.7)	18 (12.4)	
Grade III-IV	3 (10.0)	7 (4.8)	
cGVHD n (%)			0.025
No	24 (80.0)	138 (95.2)	
Mild	2 (6.7)	2 (1.4)	
Moderate	1 (3.3)	2 (1.4)	
Severe	3 (10.0)	3 (2.0)	
Severe pneumonia n (%)			0.048
Yes	8 (26.7)	16 (11.0)	
No	22 (73.3)	129 (89.0)	

PGF, poor graft function; GGF, goor graft function; AA, aplastic anemia; AL, acute leukemia; HLH, hemophagocytic lymphohistiocytosis; SF, serum ferritin; HLA, human leukocyte antigen; BM, bone marrow; UCB, umbilical cord blood; PB, peripheral blood; CMV, cytomegalovirus; EBV, Epstein-Barr virus; BKV, BK, virus; aGVHD, Acute graft-versus-host disease; cGVHD, Chronic graft-versus-host disease.

### Risk factors for PGF

Univariate analysis identified advanced transplantation age (≥10 years, P < 0.001), HLA mismatch (P = 0.018), pre-PGF CMV infection (P = 0.007), and BKV infection (P = 0.001) as significant predictors of PGF. These factors were then analyzed using multivariate logistic regression. The results showed that age ≥10 years at the time of transplantation (OR = 29.27, 95%CI: 5.70–150.21, P < 0.001), HLA mismatch (OR = 4.11, 95%CI: 1.45–11.65, P = 0.008), CMV infection (OR = 7.64, 95%CI: 2.31–25.21, P = 0.001), and BKV infection (OR = 12.22, 95%CI: 2.49–59.89, P = 0.002) were identified as independent risk factors for PGF (Table 2).

**TABLE 2:** Univariate-multivariate analysis of risk factors for PGF.

Variables	Univariate analysis for PGF		Multivariate analysis for PGF	
	Or (95%CI)	P Value	Or (95%CI)	P Value
Gender				
Male	1.00		-	
Female	1.25 (0.56–2.77)	0.581	-	
Age (years)				
<5	1.00		1.00	
≥5, <10	2.02 (0.51–8.00)	0.317	2.79 (0.56–14.02)	0.212
≥10	10.92 (2.97–40.10)	<0.001	29.27 (5.70–150.21)	<0.001
SF level, ng/mL				
<2000	1.00		-	
≥2000	1.000 (0.45–2.31)	1.000	-	
Splenomegaly				
No	1.00		-	
Yes	1.12 (0.50–2.47)	0.789	-	
Types of primary onset				
Malignant	1.00		-	
Non-malignant	0.38 (0.14–1.03)	0.057	-	
Blood mismatch				
Identical	1.00		-	
Mismatch	1.54 (0.70–3.40)	0.281	-	
HLA disparity				
Matched	1.00		1.00	
Mismatched	2.63 (1.18–5.86)	0.018	4.11 (1.45–11.65)	0.008
Source of stem cell				
BM	1.00		-	
BM + UCB	0.32 (0.06–1.62)	0.168	-	
BM + PB	0.84 (0.25–2.78)	0.773	-	
PB	7.50 (0.53–105.28)	0.135	-	
CD34^+^ cell dose, 10^6^/kg				
<5	1.00		-	
≥5, <10	0.50 (0.18–1.43)	0.198	-	
≥10	0.81 (0.33–1.99)	0.650	-	
TNC dose, 10^8^/kg				
<10	1.00			
≥10	2.178 (0.93–5.08)	0.071		
CMV infection before PGF				
No	1.00		1.00	
Yes	3.20 (1.37–7.47)	0.007	7.64 (2.31–25.21)	0.001
EBV infection before PGF				
No	1.00		-	
Yes	1.61 (0.73–3.57)	0.238	-	
BKV infection before PGF				
No	1.00		1.00	
Yes	7.17 (2.37–21.75)	0.001	12.22 (2.49–59.89)	0.002
Hemorrhagic cystitis before PGF				
No	1.00		-	
Yes	2.15 (0.97–4.77)	0.059	-	
aGVHD before PGF				
No	1.00		-	
Grade I-II	0.98 (0.27–3.65)	0.981	-	
Grade III-IV	1.41 (0.28–7.17)	0.682	-	
cGVHD before PGF				
No	1.00		-	
Mild	2.56 (0.22–29.19)	0.450	-	
Moderate	2.56 (0.22–29.19)	0.450	-	
Severe	1.70 (0.17–17.00)	0.650	-	
Severe pneumonia before PGF				
No	1.00		-	
Yes	1.61 (0.54–4.80)	0.391	-	

PGF, poor graft function; GGF, goor graft function; SF, serum ferritin; HLA, human leukocyte antigen; BM, bone marrow; UCB, umbilical cord blood; PB, peripheral blood; TNC, total nucleated cell.

CMV, cytomegalovirus; EBV, Epstein-Barr virus; BKV, BK, virus; aGVHD, Acute graft-versus-host disease; cGVHD, Chronic graft-versus-host disease.

**FIGURE 1:**
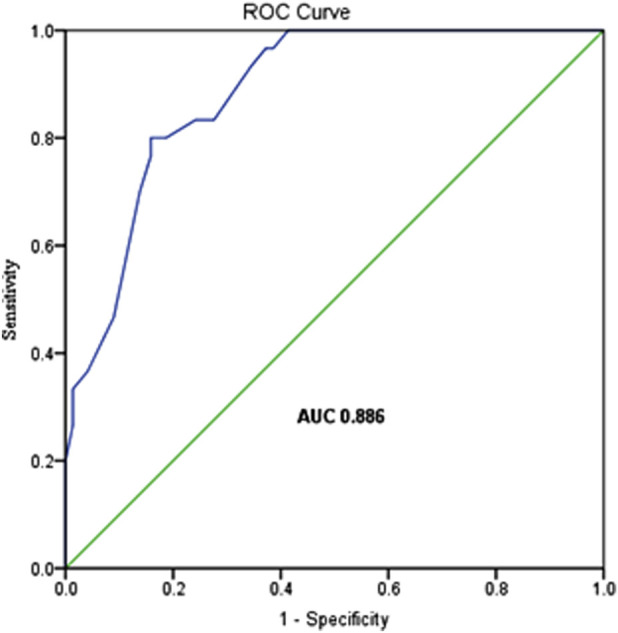
The ROC curve of the logistic regression model.

The predictive performance of the model was assessed via ROC curve analysis, yielding an area under the curve (AUC) of 0.886 (95%CI: 0.83–0.94; P < 0.001). This model exhibits a relatively high level of diagnostic accuracy, with sensitivity and specificity values of 80.0% and 84.1%, respectively, demonstrating robust utility for PGF risk stratification (Figure 1).

### Survival analyses

**FIGURE 2:**
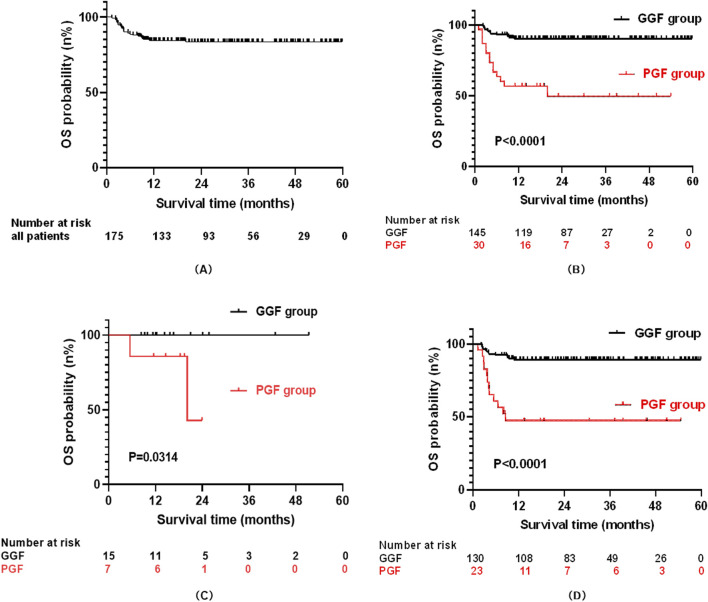
The curve for overall survival (OS): All patients **(A)**; PGF and GGF patients **(B)**; Patients with PGF and GGF in the malignant group **(C)**; Patients with PGF and GGF in the no-malignant group **(D)**.

As of 31 December 2023, the median follow-up duration for post-transplant OS was 24.97 months (range: 1.33–59.87 months). Of the 175 enrolled patients, 28 (16.0%) had died and 147 (84.0%) remained alive. The estimated 4-year OS rate for the entire cohort was 84.3% ± 2.8% (Figure 2A).

Stratified by graft function outcomes, the PGF group (n = 30) exhibited significantly poorer survival, with 14 deaths (46.7%) and 16 survivors (53.3%) at the end of follow-up, yielding a median survival of 13.5 months (range: 1.33–54.63 months). In contrast, the GGF group (n = 145) demonstrated markedly better outcomes, with 14 deaths (9.7%) and 131 survivors (90.3%). Consequently, the 4-year OS rate was substantially lower in the PGF group compared to the GGF group (49.4% ± 10.3% vs. 90.2% ± 2.5%, P < 0.001; Figure 2B).

When stratified by pre-transplant primary disease type, the cohort included 22 patients with malignant diseases and 153 patients with non-malignant diseases. By the end of follow-up, 2 deaths (9.1%) were recorded in the malignant disease group, with 20 patients (90.9%) surviving. In the non-malignant disease group, 26 deaths (17.0%) occurred, and 127 patients (83.0%) remained alive. In the malignant disease subgroup, the 2-year OS rate was significantly lower in patients with PGF than in those with GGF (42.9% ± 31.0% vs. 100%, P = 0.0314; Figure 2C). Conversely, within the non-malignant disease subgroup, the 4-year OS rate remained significantly reduced in the PGF group compared to the GGF group (47.4% ± 10.5% vs. 89.1% ± 2.7%, P < 0.001; Figure 2D).

To identify clinical factors associated with reduced OS, Cox proportional hazards regression analyses were performed. Univariate analysis revealed significant associations between decreased OS and the following risk factors: HLA mismatch (P = 0.005), PGF (P < 0.001), post-transplant EBV infection (P = 0.025), hemorrhagic cystitis (P = 0.013), aGVHD (grade I-II, P < 0.001; grade III-IV, P < 0.001), and severe pneumonia (p < 0.001). Variables with P < 0.05 in univariate analysis were subsequently included in multivariate modeling, which identified four independent predictors of diminished OS: post-transplant PGF (HR = 2.39, 95%CI: 1.02–5.59, P = 0.044), aGVHD (grade I/II, HR = 3.43, 95%CI: 1.29–9.15, P = 0.014; grade III/IV, HR = 8.92, 95%CI: 3.19–24.96, P < 0.001), hemorrhagic cystitis (HR = 3.18, 95%CI: 1.37–7.39, P = 0.007), and severe pneumonia (HR = 4.42, 95%CI: 1.92–10.19, P < 0.001). These results highlight the substantial prognostic impact of post-transplant complications, underscoring the importance of early detection and aggressive management of these conditions to optimize survival outcomes (Table 3).

**TABLE 3:** Univariate-multivariate analysis of risk factors for OS.

Variables	Univariate analysis for OS		Multivariate analysis for OS	
	HR (95%CI)	P Value	HR (95%CI)	P Value
Gender n (%)				
Male	1.00		-	
Female	0.85 (0.39–1.83)	0.673	-	
Age (years)				
<5	1.00		-	
≥5, <10	1.75 (0.62–4.97)	0.292	-	
≥10	2.61 (0.91–7.52)	0.075	-	
SF level, ng/mL				
<2000	1.00		-	
≥2000	1.18 (0.53–2.55)	0.681	-	
Types of primary onset				
Malignant	1.00		-	
Non-malignant	1.95 (0.46–8.21)	0.364	-	
Blood mismatch				
Identical	1.00		-	
Mismatch	1.29 (0.62–2.70)	0.507	-	
HLA disparity				
Matched	1.00		1.00	
Mismatched	2.90 (1.38–6.09)	0.005	1.64 (0.66–4.07)	0.288
Source of stem cell				
BM	1.00		-	
BM + UCB	0.70 (0.20–2.47)	0.574	-	
BM + PB	0.66 (0.22–1.95)	0.449	-	
PB	2.07 (0.23–18.56)	0.517	-	
CD34^+^ cell dose, 10^6^/kg				
<5	1.00		-	
≥5, <10	0.93 (0.37–2.30)	0.870	-	
≥10	0.82 (0.34–1.97)	0.649	-	
TNC dose, 10^8^/kg				
<10	1.00			
≥10	1.09 (0.52–2.31)	0.816		
PGF before OS				
No	1.00		1.00	
Yes	6.06 (2.88–12.75)	<0.001	2.39 (1.02–5.59)	0.044
CMV infection before OS				
No	1.00		-	
Yes	1.403 (0.60–3.30)	0.438	-	
EBV infection before OS				
No	1.00		1.00	
Yes	2.35 (1.11–4.97)	0.025	1.50 (0.63–3.59)	0.360
BKV infection before OS				
No	1.00		-	
Yes	2.50 (0.95–6.58)	0.064	-	
Hemorrhagic cystitis before OS				
No	1.00		1.00	
Yes	2.58 (1.22–5.46)	0.013	3.18 (1.37–7.39)	0.007
aGVHD before OS				
No	1.00			
Grade I-II	4.93 (2.01–12.08)	<0.001	3.43 (1.29–9.15)	0.014
Grade III-IV	22.58 (9.03–56.47)	<0.001	8.92 (3.19–24.96)	<0.001
cGVHD before OS				
No	1.00		-	
Mild	1.73 (0.23–12.83)	0.591	-	
Moderate	2.20 (0.30–16.32)	0.440	-	
Severe	3.02 (0.91–10.05)	0.072	-	
Severe pneumonia before OS				
No	1.00		1.00	
Yes	10.37 (4.91–21.89)	<0.001	4.42 (1.92–10.19)	<0.001

OS, over survival; PGF, poor graft function; GGF, goor graft function; SF, serum ferritin; HLA, human leukocyte antigen; BM, bone marrow; UCB, umbilical cord blood; PB, peripheral blood; TNC, total nucleated cell; CMV, cytomegalovirus; EBV, Epstein-Barr virus; BKV, BK, virus; aGVHD, Acute graft-versus-host disease; cGVHD, Chronic graft-versus-host disease.

## Discussion

Advances in transplantation protocols have improved the prevention and management of complications following allo-HSCT. Nevertheless, PGF remains a critical complication that negatively impacts outcomes in pediatric patients. Inconsistent definitions of PGF contribute to significant variability in reported incidence rates and associated risk factors. In this cohort, the overall incidence of PGF was 17.1%, consistent with the previously reported range of 5.0%–27.0% (Dominietto et al., 2001; Man et al., 2022). Primary PGF constituted 1.7% of cases, closely aligning with the 1.5% reported by Zhao et al. 2019. But markedly lower than the 5.6% observed by Sun et al. 2015. Secondary PGF occurred in 15.4% of cases, matching the findings of Zhao et al. 2019.

Higher recipient age has been established as a risk factor for PGF in prior studies (Xiao et al., 2014; Alchalby et al., 2016). In our pediatric cohort, recipients aged ≥10 years exhibited a significantly increased risk of PGF. This association may be attributable to the high prevalence of transfusion-dependent thalassemia major (67.4%) in the cohort, where older recipient typically experience cumulative transfusion burden, leading to parenchymal iron deposition. Iron overload generates reactive oxygen species (ROS) via Haber-Weiss/Fenton reactions, inducing oxidative stress that suppresses BCL2 expression and promotes erythroid apoptosis (Taoka et al., 2012). Additionally, it triggers DNA damage and CD34^+^ cell depletion in bone marrow (Ohmoto et al., 2017), potentially mediating PGF pathogenesis. However, definitive validation of this mechanism requires confirmation through large-scale prospective studies. Furthermore, our findings highlight the importance of performing HSCT before the age of 10 years in thalassemia patients, whenever clinically feasible, to mitigate iron accumulation and preempt microenvironmental damage.

With the continuous refinement of haploidentical hematopoietic stem cell transplantation protocols, pediatric patients undergoing this approach can achieve long-term outcomes comparable to those of matched sibling donor HSCT (Lv et al., 2019). However, a subset of patients still experience PGF following hematopoietic reconstitution, which poses a significant threat to long-term survival. In our study, HLA mismatch was identified as an independent risk factor for PGF development, consistent with previous findings (Sun et al., 2019; Lv et al., 2021; Alchalby et al., 2016). Nevertheless, the precise mechanisms by which HLA disparity mediates PGF remain incompletely elucidated, warranting further exploration and validation through additional clinical research.

CMV infection following allo-HSCT can involve critical organs and increase the risk of PGF and GVHD, which is closely associated with the immune reconstitution of CMV-specific T cells (Reddehase et al., 2021; Degli-Esposti and Hill, 2022). During the first year post-transplantation, clonal expansion of CMV-specific effector memory T cells drives this process. Our analyses, consistent with prior retrospective studies, demonstrated CMV reactivation as an independent risk factor of PGF in both univariate and multivariate models (Lv et al., 2021; Lin et al., 2022). However, conflicting data exist; another study identified CMV infection as a risk factor for primary PGF in univariate analysis, but this association did not persist after multivariate adjustment (Zhao et al., 2019). These discrepant findings suggest that whether CMV infection constitutes a risk factor for PGF remains controversial, underscoring the need for rigorous validation through well-designed prospective multicenter cohort studies.

BKV typically remains latent in immunocompetent individuals, with primary infections often asymptomatic. In pediatric allo-HSCT recipients, however, treatment-induced immunosuppression frequently reactivates latent BKV, leading to clinical complications. While BKV is a well-known cause of post-transplant hemorrhagic cystitis (McCaffrey et al., 2021; Janeczko-Czarnecka et al., 2020), its role in other allo-HSCT-related outcomes remains poorly understood. This study identifies BKV reactivation as an independent risk factor of PGF, extending its clinical relevance beyond hemorrhagic cystitis. These findings suggest that early BKV monitoring and preemptive therapy during the post-transplant period may reduce PGF incidence. However, the causal relationship between the reactivation of latent viruses (such as CMV and BKV) and the occurrence of PGF remains controversial, as PGF can lead to delayed hematopoietic and immune reconstitution, thereby increasing susceptibility to viral infections. Definitive establishment of causality requires large-scale, multi-center prospective cohort studies incorporating protocol-based virological surveillance and rigorously standardized clinical endpoints.

Furthermore, the severity of the underlying disease may substantially increase the risk of PGF through multiple synergistic pathways, including direct impairment of hematopoietic stem cell and microenvironmental reserves. Concurrently, high-intensity pre-transplant conditioning regimens may exacerbate PGF risk via direct cytotoxic effects and immune dysregulation. Future studies should incorporate detailed stratification based on disease risk indices and conditioning intensity, to better isolate independent predictors of PGF.

Patients who develop PGF following transplantation are associated with a poor prognosis. In this study, the 4-year OS was significantly lower in the PGF group compared to the GGF group among pediatric recipients, consistent with prior reports (Zhao et al., 2019; Sun et al., 2019; Prabahran et al., 2021). However, the management of PGF remains investigational. Currently, therapeutic strategies for PGF are primarily developed based on its underlying pathological mechanisms. These include interventions such as second donor stem cell infusion, purified CD34^+^ cell infusion, MSC transfusion, and thrombopoietin receptor agonists (e.g., eltrombopag) (Shahzad et al., 2021; Servais et al., 2023; Mahat et al., 2020). Univariate-multivariate analysis in this study identified PGF as an independent risk factor for reduced OS, alongside hemorrhagic cystitis, grade I-II aGVHD, grade III-IV aGVHD, and severe pneumonia. These findings highlight the critical need to mitigate PGF occurrence through targeted preventive strategies, which may substantially improve survival outcomes in this vulnerable population.

In conclusion, PGF is characterized by high incidence and poor prognosis. This study demonstrates that PGF is an independent risk factor for reduced OS, underscoring the imperative to mitigate PGF occurrence as a key strategy to improve clinical outcomes. The study also identified recipient age ≥10 years, HLA mismatching, and post-transplant CMV or BKV infections as independent risk factors for PGF after allo-HSCT in pediatric. Optimizing transplantation protocols to address these risk factors—such as performing allo-HSCT at a younger age, improving HLA compatibility, and enhancing antiviral prophylaxis—may reduce PGF incidence. Furthermore, investigating early predictive biomarkers for PGF could guide timely clinical interventions, enabling proactive management to enhance OS in affected patients.

However, this study has several limitations. First, as a retrospective investigation, it may be subject to selection bias due to the exclusion of cases with incomplete information. Second, the sample size from a single center was insufficient to develop separate risk models for primary and secondary PGF. Future multi-center prospective studies are warranted to further elucidate the mechanisms underlying PGF and to identify predictive biomarkers for its occurrence, thereby providing a basis for early clinical intervention and treatment.

## Data Availability

The original contributions presented in the study are included in the article/Supplementary Material, further inquiries can be directed to the corresponding authors.
