# Supplementary material for: Risk factors and prognosis of poor graft function after allogeneic hematopoietic stem cell transplantation in pediatric: a retrospective study
Source: Front Cell Dev Biol. 2025 Oct 8;13:1651658. doi: 10.3389/fcell.2025.1651658 (PMC12540324; doi:10.3389/fcell.2025.1651658)
Supplement: Supplementary file 1 [file Table1.docx]

**Supplementary material**

All pediatric patients underwent pre-transplant conditioning regimens to eradicate abnormal clonal cells and disrupt the underlying disease pathogenesis. In this study, the conditioning regimens administered to the 175 pediatric patients were as follows:

Regimen 1: Busulfan (BU) + Cyclophosphamide (CY) + Rabbit Anti-Human Thymocyte Globulin (ATG) + Fludarabine (Flu) (n=143)

Regimen 2: Cytarabine (Ara-C) + BU + CY + ATG + Lomustine (n=5)

Regimen 3: Ara-C + BU + Thiotepa (TT) + CY + ATG (n=2)

Regimen 4: Ara-C + BU + CY + ATG (n=8)

Regimen 5: BU + CY + ATG + Etoposide (VP-16) + Melphalan (n=6)

Regimen 6: BU + CY + Melphalan (n=3)

Regimen 7: BU + CY + ATG +Flu + Melphalan (n=3)

Regimen 8: BU + CY + ATG + Flu + VP-16 + Melphalan (n=3)

Regimen 9: BU + CY + ATG + Flu + Etoposide (VP-16) (n=1)

Regimen 10: BU)+ CY + ATG + Flu + Methotrexate (MTX) (n=1)
